# Restoration of insect communities after land use change is shaped by plant diversity: a case study on carabid beetles (*Carabidae*)

**DOI:** 10.1038/s41598-023-28628-7

**Published:** 2023-02-07

**Authors:** Markus Lange, Anne Ebeling, Winfried Voigt, Wolfgang Weisser

**Affiliations:** 1 Max Planck Institute for Biogeochemistry, Jena, Germany; 2 Institute of Ecology and Evolution, University of Jena, Jena, Germany; 3 Terrestrial Ecology Research Group, Department of Life Science Systems, School of Life Sciences, Technical University of Munich, Freising, Germany

**Keywords:** Biodiversity, Restoration ecology, Grassland ecology

## Abstract

There is no doubt about the insect decline currently taking place in ecosystems with large anthropogenic impacts. Thus, there is a need for practices that avoid insect decline and or help to recover insect communities that have already suffered. Plant diversity has been shown to be positively related to insect abundance and diversity and to ecosystem functions provided by insects. However, it remains open if increased plant diversity can help to recover decreased populations. Here, we tested over one decade the effects of plant diversity on the carabid community in a large grassland biodiversity experiment and how plant diversity fostered the establishment of a natural grassland community after conversion of an arable field. There was a dramatic decline in carabid abundance from 2003, the first year after establishing the diversity experiment, to 2005. However, subsequently, the abundance increased constantly. One year after the land use change most individuals and species were those commonly found in agricultural fields. In subsequent years the community was dominated by grassland species. While plant diversity did not affect the abundance and richness of the carabid community, the turnover to a more native grassland community was accelerated by plant diversity in the first years after the land use change. In contrast, in later years plant diversity stabilized the community assemblage. Our study shows that high plant diversity can contribute to a faster transition of insect populations towards naturally occurring community assemblages and at later stages to more stabilized assemblages.

## Introduction

There is no doubt about the worldwide reduction of natural habitats due to human land use change and land use intensification, causing a dramatic loss of species diversity across taxonomic groups^1–4^. Insects are particularly affected by human impacts^4–7^, with severe consequences for ecosystem functioning^8^. Changes in insect communities — in terms of their diversity, biomass or composition — also entail changes in e.g. food web dynamics^9^ and thus pollination^10^, natural pest control^11^, pathogen damage or decomposition. To counteract the decline in insects, practices need to be developed that help to recover or stabilize formerly degraded insect communities.

Increasing local plant diversity could be such a strategy to promote insect communities, as plant diversity was shown to increase insect diversity, abundance, biomass or trait diversity^12–16^, and consequently, insect-mediated ecosystem functions^8,10,11,17^. In addition to the increase in food resource diversity, an increase in the number of plant species lead to higher productivity^18,19^, altered habitat structure, and microclimatic conditions, all of those factors are potentially important for an enrichment of insect communities^20–23^. Specifically, due to strong bottom up effects, the increase in plant resource diversity not only enhances the attractiveness for specialized herbivores, but also for carnivores feeding on them^24–28^. Similarly, increased habitat complexity driven by an increase in plant diversity benefits not only individual insect species, but might affect all species that are directly or indirectly connected with them. However, if and how plant diversity contributes to the restoration of insect communities that were formed by arable land use has not yet been studied in an experimental context.

In studies on habitat-insect relationships, carabid or ground beetles often play an important role for several reasons. First of all, they are relatively abundant and species-rich in grasslands and arable fields^29^, making them a suitable organism group for comparative studies. Second, due to their sensitivity to habitat structure and microclimate^23,30,31^ carabid beetles are often used as a bioindicator and to assess habitat quality^32^. Third, carabids play an important role in food webs. Although there are a number of trophic specializations among them, they are generally considered polyphagous predators^32^, therefore affecting lower trophic levels (e.g. herbivores and decomposers) and associated ecosystem functions^17,33^. In this role, they are important for e.g. natural pest control in arable land^34,35^, and might strongly influence pathways of energy and material flows^8,36,37^. Lastly, during the last decades, human impact has led to a strong decrease in carabid biomass and species richness, reviewed in Holland and Luff^38^, making it important to examine the consequences of possible restoration efforts for this threatened insect group.

Here we studied how carabid communities evolve following a land use change from arable land to grassland, and if the diversity of the grassland affects the transition processes. We took advantage of one of the world’s longest-running biodiversity experiment, the Jena Experiment^39^. For a period of one decade we investigated how the carabid beetle community was shaped by plant species richness, after the experiment was set up on a former arable field. Specifically, we asked the following questions: (i) Is there a change in abundance and species richness of carabids within the first ten years after land-use conversion from arable field to an experimental grassland? (ii) Do carabid communities in the established experimental grasslands benefit from an increase in plant species richness (higher abundance and species richness) and does it have an effect on the carabid community assemblage (arable land versus grassland species)? (iii) Does the carabid community assemblage change over time (arable land versus grassland species), and if yes, is this driving the strength and direction of the plant species richness- carabid community (species richness, abundance, assemblage) relationship?

## Material and methods

### Study site

The study was carried out in the Jena Experiment, a large-scale grassland diversity experiment in the floodplain of the Saale River near the city of Jena Thuringia, Germany; 50°57´N, 11°35´E^39,40^. In spring 2002, 82 experimental grassland plots of 20 × 20 m were established. Plots are arranged in four blocks to account for changes in soil characteristics with increasing distance from the river, typical for a flood plain. Specifically, soil texture in the upper soil ranges from sandy loam to silty clay with increasing distance from the river. Sand content declines from 40% near the river to 7% at the furthest plot, while silt content increases from 44 to 69%, respectively. The clay content ranges from 16 to 24%, but is not related to distance from the river. The experimental field site of this study was an arable field with mineral fertilizer input to grow wheat and vegetables for about 40 years before the establishment of the Jena grassland experiment. After the last harvest in autumn 2000 the field was ploughed and kept fallow throughout 2001. In preparation for the experiment, and in order to reduce the weed pressure the field was harrowed bimonthly (June, August, October) and treated with Glyphosate (N-(Phosphonomethyl)-glycin, Roundup) in July 2001. In spring 2002 the field was harrowed before the plots were established^39^.

**Figure 1:**
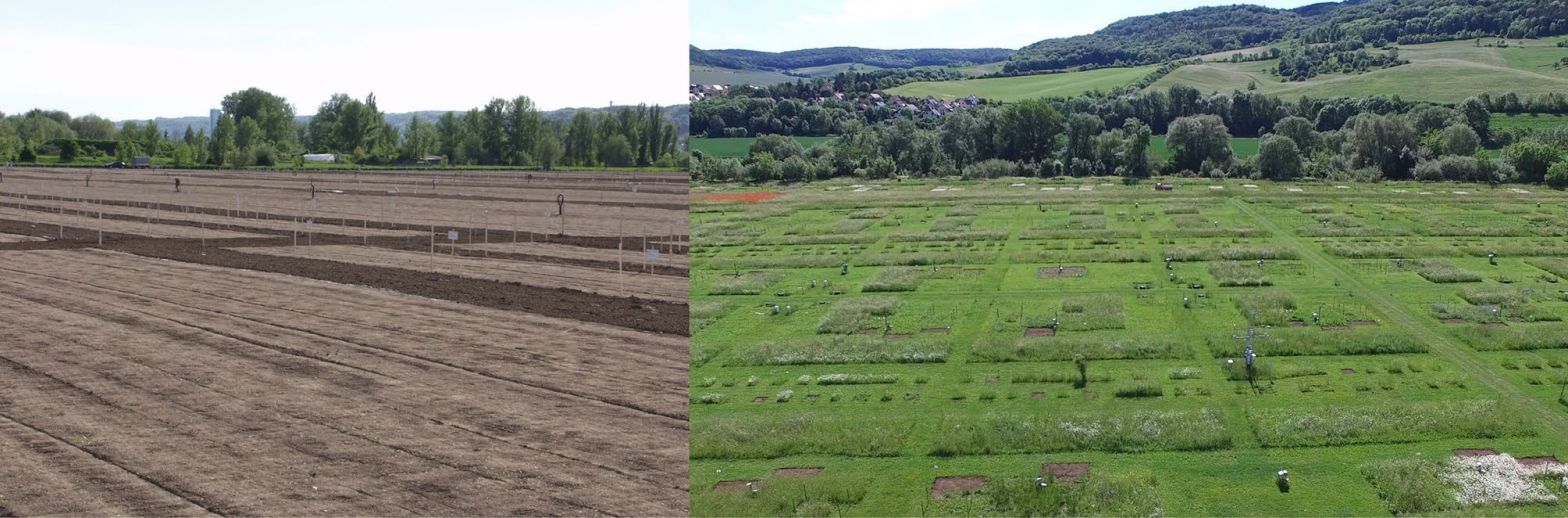
The 10-ha site was used as arable land until 2000, and was converted to an experimental grassland in 2002. For this, the area was completely tilled and experimental plots of different plant diversity were sown, separated from each other by paths (left photo: field site in 2002; right photo: field site in 2016). The plant community composition of each plot was randomly selected out of a species pool of 60 grassland species. Each diversity level is replicated 16 times, except for 16 and 60-species mixtures, which have 14 and 4 replicates, respectively. After the plots became established, colonization by above- and belowground fauna began. Left photo by K. Kübler, copyright of right photo by the Jena Experiment.

Plant communities of different plant species richness (PSR) were established building a gradient from monocultures to 60 plant species mixtures (diversity levels: 1, 2, 4, 8, 16 and 60 species). The plant diversity levels were replicated 16 times, except for the 16 (14 replicates) and 60 (four replicates) plant species mixtures (Fig. 1).

The species were randomly chosen from a pool of 60 Arrhenatherion grassland plant species and from four functional groups, namely grasses, legumes, small herbs, and tall herbs (Table S1). Functional group classification was based on morphological, phenological, and physiological traits^39^. For this study, the PSR levels 1, 4, 16 and 60 were used, resulting in fifty plots. Experimental plots are weeded manually two to three times a year to maintain the target plant community composition. Weeds were mainly species of the species pool that are not sown on the respective plot. The plots are mowed and the mowed plant material is removed twice a year in June and September, but not fertilized, which is typical for extensively managed hay meadows in Central Europe. In 2009 the original plot size was reduced to 105 m^2^ ^40^.

### Carabid sampling

In 2003, 2005, 2010 and 2012 two pitfall traps, cup-traps with a diameter of 4.5 cm and filled to one third height with 3–5% formalin with some drops of detergent, were installed in the center of each of the 50 plots from the beginning of May to mid-October. Except for the period immediately after mowing when traps were closed for about two weeks, the traps were each emptied after a period of three weeks in the first two years of sampling and fortnightly in the last two years of sampling. This resulted in similar sampling days per year (2003 = 140 d, 2005 = 142 d, 2010 = 123 d, 2012 = 135 d). Additionally, carabid communities were sampled in 2005, 2010, and 2012 in semi-natural meadows adjacent to the experimental field.

All arthropods were sorted and the ground beetles were identified to species level by a designated specialist using the keys by Freude et al.^41^. Carabid species were divided into three groups due to their preferences for habitat types, based on the information obtained from literature of carabid ecology^42^. The following grouping was used with respect to habitat types: meadow and lawn species, arable land species, and species with no or other special habitat preference. The latter group also included less frequent groups such as riparian species. A species was assigned to the meadow and grassland group if its occurrence on meadows or grasslands is described in the literature as obligate or optional. The group of arable land only included species for which this habitat type was described as obligate or with a strong preference. For some species, not a habitat type but a preference to abiotic factors is described as a preference for a particular soil texture or humidity. These species were assigned to other habitat preferences. The assignment of species to the different habitat groups and the total number of samples per year is given in Table S2.

### Data analysis

The carabid data of all sampling times across each year were pooled for statistical analysis. Total carabid abundance (N), the species richness of the entire carabid community (S), the proportional abundance and richness of grassland and arable land species, as well as the Evenness (calculated as Shannon diversity dividend by the log-transformed species richness) were assessed to investigate if the carabid community (i) changed over time, (ii) if there were effects of plant diversity and (iii) if the carabid community assemblage and possible plant diversity effects changed over time.

All analyses were conducted in R^43^. Linear Mixed-Effects Models (LMM) were employed using the ‘lmer’-function in the ‘lme4’ package^44^. Starting from a constant null model with plot as random intercept, to account for the repeated measurements over the years, the null model was stepwise extended. Block was fitted as fixed effect because it does not fulfill the requirement of a random sample with normally distributed effects since they are systematically arranged in a linear sequence^45^. The fitting sequence of fixed terms followed the order: block, year (as factor), plant species richness (PSR, log-transformed) and the year × PSR interaction. The maximum likelihood method (ML) was applied and likelihood ratio tests (Chi^2^ ratio) were used to compare succeeding models and test for a significant model improvement by the added fixed effects^46^. The results of the full analysis models were reported. Plant species richness was log-transformed to achieve linearity and to obtain a more normal distributed error structure and to stabilize variances. Differences among years were assessed based on estimated marginal means (EMM) using the ‘emmeans’-function of the ‘emmeans’ package^47^. Please see Supplementary Information (SI) for the R markdown, including all statistical tests.

To compare carabid community composition and their development over time a Principal Coordinates Analysis (PCoA) was applied using the ‘cmdscale’-function in the ‘vegan’ package^48^. Prior to this analysis the carabid community was Hellinger transformed (‘decostand’-function, ‘vegan’ package) and the distance matrix was calculated based on the Bray–Curtis distance (‘vegdist’-function, ‘vegan’ package). To assess the impact of plant species richness on the community composition within and across years Permutational Multivariate Analysis of Variance (PERMANOVA; based on the Bray–Curtis dissimilarity) was performed applying the ‘adonis’-function (permutations = 999) in the ‘vegan’ package with the same fitting sequence as describe for the LMM. For a quantitative assessment of the temporal turnover of the carabid communities, the Bray–Curtis distance of each plot over time (2003–2005, 2005–2010, 2010–2012) was used. The effects of plant species richness on turnover were tested by applying a model structure similar to that of the LLM (SI).

## Results

**Figure 2:**
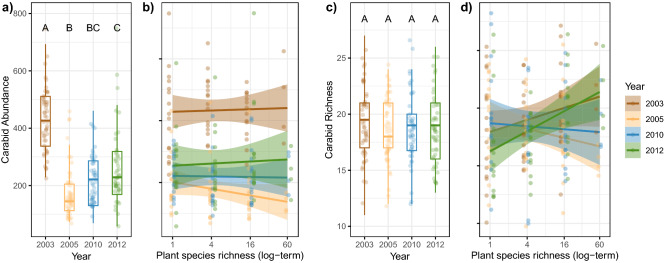
Numbers of (**a**) individuals (Abundance) and (**c**) species (Richness) of carabid beetles at different years and (**b**), (**d**) as affected by plant species richness. Lines in boxes represent median, top and bottom of boxes represent first and third quartiles, and whiskers represent 1.5 interquartile range. Differences among the years are assessed based on estimated marginal means. Boxes with the same letters are not statistically different. Bands in regression plots represent the level of confidence interval (0.95). Dots represent partial residuals for Year and Plant species richness.

The total number of carabid individuals captured in two traps per plot over a period of five months per year decreased strongly from 2003 (433.4 individuals ± 122.9 S.D.) to 2005 (177.1 ± 95.7) and increased again in 2010 (219.3 ± 95.5) and 2012 (262.3 ± 146.5), but only to about half the number in 2003. While carabid abundance was highest in 2003, carabid richness was relatively stable among the years (2003: 19.3 ± 3.4, 2005: 18.5 ± 2.9, 2010: 18.7 ± 3.6, 2012: 18.6 ± 3.3, Fig. 2).

### Effects of plant species richness on carabid abundance and richness over time

Overall, carabid abundance and richness were not affected by plant species richness. For total carabid abundance this was consistent for all years observed. In contrast, the significant interaction-term Year × PSR for carabid richness indicated different plant species richness effects at different years (Table 1, SI). However, these differences did not follow a temporal trend (Fig. 2).

**Table 1:** Results of linear mixed effects models (LMM) testing the effects of plant species richness (PSR) and its changing effect over time on carabid abundance, richness, the proportion (%) of arable and grassland individuals and species and on the turnover of the community assemblage.

	Block		Year		PSR		Year x PSR	
	Chi^2^	*P* value	Chi^2^	*P* value	Chi^2^	*P* value	Chi^2^	*P* value
Abundance (total)	2.55		136.42	***	0.10		2.40	
Richness (total)	7.36		1.90		2.05		12.20	**
Abundance grassland (%)	4.16		277.91	***	0.16		7.49	
Richness grassland (%)	0.46		3.73		2.67		9.94	*
Evenness grassland	1.42		44.91	***	0.03		21.47	***
Abundance arable land (%)	2.29		396.66	***	0.01		4.84	
Richness arable land (%)	1.82		97.53	***	8.33	**	8.26	*
Evenness arable land	1.05		19.71	***	0.47		3.59	
Turnover (Bray–Curtis dissimilarity)	4.12		75.33	***	0.77		10.05	**

Asterisks mark the level of significance (‘***’ < 0.001, ‘**’ < 0.01, ‘*’ < 0.05, ‘.’ < 0.1). Please note, for assessing the turnover, Period was fitted instead of Year, see Methods.

### Changes in community assembly due to habitat preferences over time

**Figure 3:**
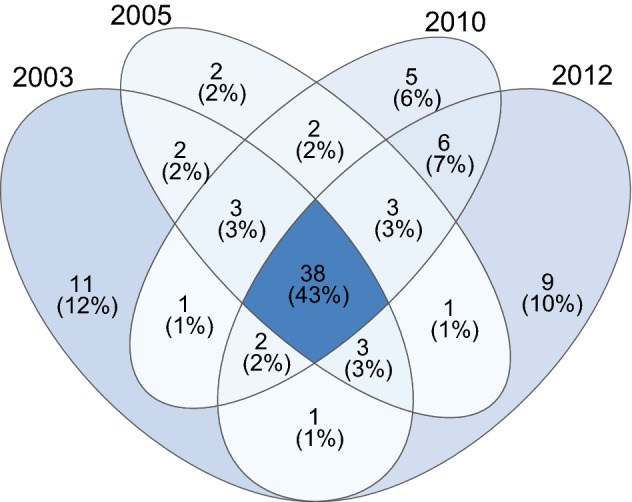
Venn diagram showing the species number occurring in individual years of the sampling and the species numbers, which were found in different years.

A large proportion of the species occurred in all years (38 species, corresponding to 43%, Fig. 3), while 12% of all species were only found in the first year of the sampling. Within the proportion of the species found in all years, the vast majority (71%) were grassland species. After the first sampling in 2003, the number of species exclusively occurring in a specific year increased with time from 2% in 2005 up to 10% in 2012 (7% in 2010). Moreover, species found only in one sampling year occurred on very few abundances, mostly less than five individuals (Table S2). These rare species were also mostly grassland species (50%) and species with other habitat preferences, such as riparian species (46%). In 2005 one arable land species, two grassland species and five species with other habitat preferences were recorded for the first time. They were mostly sampled with only one or two individuals, so that their relative abundance was very low with 0.5% (Table S2). In 2010 one arable, four grassland species and six species with other habitat preferences were newly recorded. Their relative abundance was also very low with 0.5%. In 2012, six grassland species and three species with other habitat preferences were newly recorded. Their relative abundance of 0.1% was even lower than that of the newly recorded species in previous years. Most of the newly found species remained at a relatively low abundance in the following years, only the abundance of *Microlestes maurus*, firstly recorded in 2005, and *Ophonus schaubergerianus*, firstly recorded in 2010, became abundant in the following years.

**Figure 4:**
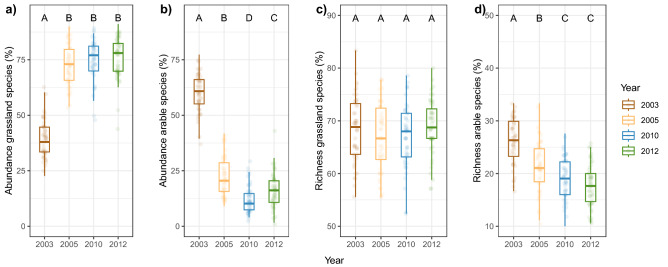
Percentage of (**a**), (**b**) abundance and (**c**), (**d**) species richness of carabid beetles assigned to grassland and arable land species at different years. Lines in boxes represent median, top and bottom of boxes represent first and third quartiles, and whiskers represent 1.5 interquartile range. Differences among the years are assessed based on estimated marginal means. Boxes with the same letters are not statistically different. Dots represent partial residuals for Year and Plant species richness. Please note the different scaling of the y-axes.

In 2003, two-thirds of all individuals and one-third of the species were classified as arable land species (Fig. 4, Table S2). On the contrary to that, meadow and grassland species contributed nearly 60% of the community’s species, but only one-third of all individuals. The abundance proportion of arable land species strongly decreased from 2003 to 2005 and remained on this low level in the subsequent years (Fig. 4). The abundance proportion of grassland species showed the opposite pattern and increased from 2003 to 2005 and remained at this high level in the following years (Table 1, Fig. 4). Furthermore, the richness proportion of grassland species did not differ across the years, but the richness proportion of the arable species constantly decreased with time. The evenness of the grassland species was generally higher than that of arable species, and from the grassland species it even increased with time while the opposite pattern was found for arable species (Fig. S1).

**Figure 5:**
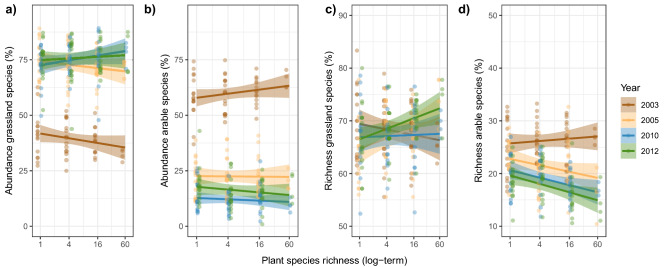
Percentage of (**a**), (**b**) carabid abundance and (**c**), (**d**) species richness of grassland and arable land carabid beetles assigned to grassland and arable land species as affected by plant species richness at different years. Bands in regression plots represent the level of confidence interval (0.95). Dots represent partial residuals for Year and Plant species richness. Please note the different scaling of the y-axes.

Plant diversity had neither an effect on the relative abundance of the grassland and arable land species nor on the relative number of species of the grassland carabids. In contrast, there was a negative plant diversity effect on the richness proportion of the arable land species except in the first year of the sampling, in 2003 (Table 1, Fig. 5). The plant diversity effect on the grassland species differed among the years (Table 1); plant diversity positively impacted the evenness of grassland species in the first two sampling years, while in the later years it was negatively affected. On the contrary, plant diversity had no effect on the evenness of arable land species (Fig. S1).

### Effect of plant diversity on carabid community composition and turnover

**Figure 6:**
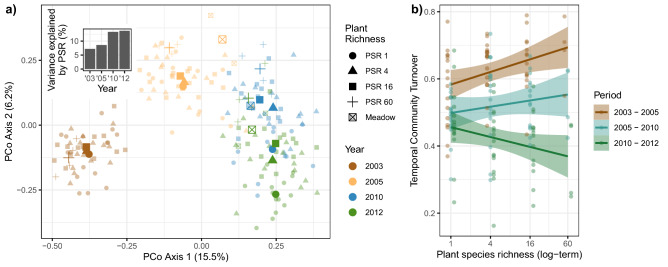
Carabid community composition and its temporal turnover: (**a**) Principal Coordinates Analysis (PCoA) based on species abundances in each plot in each year. The bar graph within this ordination plot gives the explained variance of the community by plant species richness (PSR) on an annual basis (see SI). Please note in the PCoA plot the carabid communities sampled in adjacent meadows that serve as reference. (**b**) Temporal community turnover, calculated from species abundances in a plot in two adjacent years, as a function of plant species richness. Bands represent the level of confidence interval (0.95). Small symbols in (**a**) represent single observations and large symbols the centroid per Plant species richness in each Year and in (**b**) dots represent partial residuals for Year and Plant species richness.

Carabid beetle community composition differed strongly between years (Table 2, Fig. 6a). Plant diversity impacted the community composition, but differently in the sampling years as indicated by the significant interaction term Year × PSR (Table 2). This difference was revealed when analyzing the carabid communities per year: the variance explained by plant species richness increased with time from 7.2% in 2003 to 13.7% in 2012 (Fig. 6, SI).

**Table 2:** Results of permutational multivariate analysis of variance (PERMANOVA, based on Bray–Curtis dissimilarity) testing the effects of plant species richness (PSR) and its changing effect over time on the carabid community composition, based on the Bray–Curtis dissimilarity.

	Block		Year		PSR		Year x PSR	
	F value	*P* value	F value	*P* value	F value	*P* value	F value	*P* value
Community composition	3.6	***	76.2	***	10.8	***	3.6	***

Asterisks mark the level of significance (‘***’ < 0.001).

The turnover of carabid communities differed strongly among time periods (Table 2), being highest in the first period and decreasing progressively in subsequent periods (Fig. 6b). Moreover, the turnover within the plots was not significantly affected by plant species richness (Table 2), however, the plant diversity effect differed with time (significant interaction term Period × PSR). In the first and second observation period (2003–2005, 2005–2010), the turnover of the carabid communities increased with plant species richness, i.e. changes in the carabid assemblage occured faster when plant species richness was higher. In contrast, from 2010 to 2012, turnover was lower in the more plant-diverse plots. Moreover, in the two later years the carabid communities of control meadows were much more similar to the communities of the experimental site than in 2005 (Fig. 6b), suggesting that in the later years a typical grassland community established.

## Discussion

We studied successional processes of carabid communities in grasslands of different plant species richness after conversion from an arable field. Within a decade, we found remarkable dynamics in the carabid communities including strong legacy effects. Specifically, one year after establishing the experimental grassland the carabid community was still dominated by arable land species. Beside these strong community changes over time, plant species richness influenced both, carabid community composition, with more diverse plant communities hosting more grassland-associated communities, and the rate at which the community transformed into a common grassland community. Interestingly, the plant diversity effects change over time, becoming stronger with prolongation of the experiment or changing its direction, as discussed below.

### Impacts of the land use change and legacy of agricultural use

The mean abundance of about 450 individuals per plot in 2003, one year after setting up the grassland experiment, and three years after the field was last used as an arable field, is quite high considering that we used only two pitfall traps per plot. The species community at that time was still largely dominated by a few species common in arable lands (Figs. 4, 5, Table S2), even though it was already the second grassland season. In temperate zones, the life cycle of most carabid species is annual, but some species live even more than one year reviewed in Holland and Luff^38^ and Lovei and Sunderland^49^. Thus, the dominance of arable carabids in the first year of our investigations indicated that the arable species were less affected by the immediate land use change. However, after the plant communities have become well established in their biomass production and vegtation structure after a few years^50^, the arable carabid beetles became significantly less in abundance and richness.

Establishing an experimental grassland with up to 60 different plant species was a substantial land use change in the microhabitat properties and food resource availability. Root^51 ^predicted that increasing plant species richness, in our case the change from a monoculture to a diverse grassland habitat, should have led to a higher predator abundance in the more diverse plant stands. However, this hypothesis does not fully consider disturbances, such as a change in land use practice or a complete change of plant species. Fluctuations in temperature and humidity are more extreme in arable fields than in meadows and grasslands, at least until the development of a crop canopy and after harvesting. This leads to a steeper dominance structure within the assemblage, *i.e.* only a few species are very abundant and predominating^38,52^, resulting in less even communities as found in our study (Fig. S1). These species are less affected by the agricultural practices and have a strong dispersal power^38^. In contrast to many agricultural fields, grassland assemblages are more even and dominated by a large number of species^53^.

The dramatic decrease of abundances between 2003 and 2005, i.e. between the second and fourth year after transition to a grassland, affected the majority of carabid species, but was far stronger for arable species than for grassland species, with 85% and 20% losses in abundance, respectively (Table S2). Already three years after the land use change grassland species dominated the carabid communities, as also found by Purtauf et al.^54^, who studied managed grasslands after conversion from arable lands. Moreover, in line with this study reporting that many arable species vanished in the first and second year after the conversion to grassland, we found that several species exclusively occurred in the first year of our observation. One possible reason could be that the land use change to grassland dramatically reduced resources, such as aphid pests in arable fields. In addition, the extremely warm summer of 2003, compared to the other sampling years (Table S3), may have contributed to the high first year abundance for the entire community, as the pitfall trapping methods reflects activity densities of epigeic predators^29^. The activity is likely to increase with the number of warm and hot days during the growing period. However, some arable carabid species originates from riparian habitats and were originally found on naturally formed raw soils, such as in river floodplains and their succession^49^, therefore, many carabid species have the potential to live in both, arable land and floodplain meadows, such as our study site. In addition, the abundance of phytophagous ground beetles increased (Table S2), indicating that herbivores benefited from the land use change to grassland and the increase in plant species richness compared to arable land as shown earlier by Harvey et al.^30^.

### Plant diversity effects and the turnover of the carabid community

After the severe decline of the carabid abundance in the first years after land conversion, the number of beetles increased in the following years, though without reaching the individual numbers in the first sampling year. In contrast, the number of carabid species was among all years in a similar range. Plant species richness did not impact carabid abundance and the carabid diversity was inconsistently affected during the study.

The strong increase in the ratio of grasslands-to-arable species over time, both on an abundance and species identity basis, demonstrated change and adaptation of the carabid community to the land use change from arable land to a semi-natural grassland. The positive effect of plant diversity on the increase of grassland species richness and the decrease of arable species richness furthermore indicated that higher plant diversity accelerates the change in carabid community structure towards a grassland community. In our study, land use change is accompanied by changes in plant species richness and composition, and thus inevitably by changes in vegetation structure. In addition, plots with higher plant species richness might be more diverse in their structure, allowing more grassland species to coexist but at the same time inhibiting the locomotive activities and reproduction of arable species.

At the moment, the question of whether plant diversity effects change over time is subject of intensive investigations^55^. A strengthening of plant diversity effects over time could be reported for plant related ecosystem functions like productivity^56,57^, but so far not for insect communities. For the carabid community composition in our study, the strengthening of the plant species richness effects indicate that community assembly may take several years, even for mobile, aboveground-feeding species, which emphasizes the importance of long-term studies.

This delayed and increasing response of the carabid community to the new environmental conditions is most likely caused by dispersal and life-cycle, in which the larval stage is less mobile^32^. Surprisingly, only in the first two periods the plot-specific turnover of the ground beetle assemblages was higher in plots with higher plant species richness. In the last period, plant species richness decreased the plot-specific turnover. This indicated two divergent effects taking place at different development stages of the ecosystem; while firstly plant diversity increased the turnover towards a grassland community after the land use change when the community is still adapting to the new habitat conditions, the community turnover is lower at later ecosystem stages. This indicates that high plant diversity stabilizes the community assemblage if the community is well adapted. For plant biomass production it has been shown that plant diversity stabilizes the productivity^58^. Thus, it is likely that the more stable plant production cascades up in higher trophic level and in addition, that more diverse plant communities provide more stable habitat conditions.

#### Implications

Our study confirms that insect communities changed tremendously after a land use change. In the case of ground beetles, this is independent of whether the land use change is considered towards more natural conditions with less intense anthropogenic influences on the ecosystem. This is because among carabid beetles, in contrast to other groups e.g. pollinators, there are several species adapted to highly intensely managed land, such as arable fields. These arable species will first strongly decline, until they likely vanish. However, as arable land use is assumed as one of the major causes of insect decline^5,6^, there is also a need to establish practices that diminish the insect decline and or helps to recover insect communities that have been suffering from insect decline.

Our results clearly show not only that the carabid community constantly developed after a land use change. Beside this strong temporal change of the community composition our results also demonstrate that plant species richness has a positive effect for the transition of the carabid beetle assembly typical for the newly established grassland. But the process of community assembly after starting a biodiversity experiment in the field takes considerable time, which has largely been neglected in biodiversity experiments. Our results caution against short-term experimental studies of such groups that depend on natural community assembly after the manipulation of the habitat. Blake, et al.^59^ suggested for carabid beetles the recolonization process is likely to take considerably longer than five years. Our study is in line with these previous results, however, we found plant diversity first accelerated the transition of the carabid assemblage towards the natural grassland community and then it stabilized the community assemblage. Thus, a high plant diversity accompanied with low intense land management offers on the one hand a strategy for an accelerated transition towards natural insect populations. On the other hand, many arable carabid species need non-arable habitats for reproduction and overwintering^60^. Thus, grassland habitats with a high diversity, providing stable environmental conditions, may therefore serve as temporal refuge^61^ for species that potentially contribute to pest control by spilling over into croplands^35,38^.

## Supplementary Information

Supplementary Information 1.

Supplementary Information 2.

## Data Availability

The dataset used in our analyses is available from Edmond, the Open Research Data Repository of the Max Planck Society, under 10.17617/3.JNR97G.
