# Supplementary material for: Restoration of insect communities after land use change is shaped by plant diversity: a case study on carabid beetles (Carabidae)
Source: Sci Rep. 2023 Feb 7;13:2140. doi: 10.1038/s41598-023-28628-7 (PMC9905558; doi:10.1038/s41598-023-28628-7)
Supplement: Supplementary file 1 — Supplementary Information 1. [file 41598_2023_28628_MOESM1_ESM.pdf]

Supplementary Data

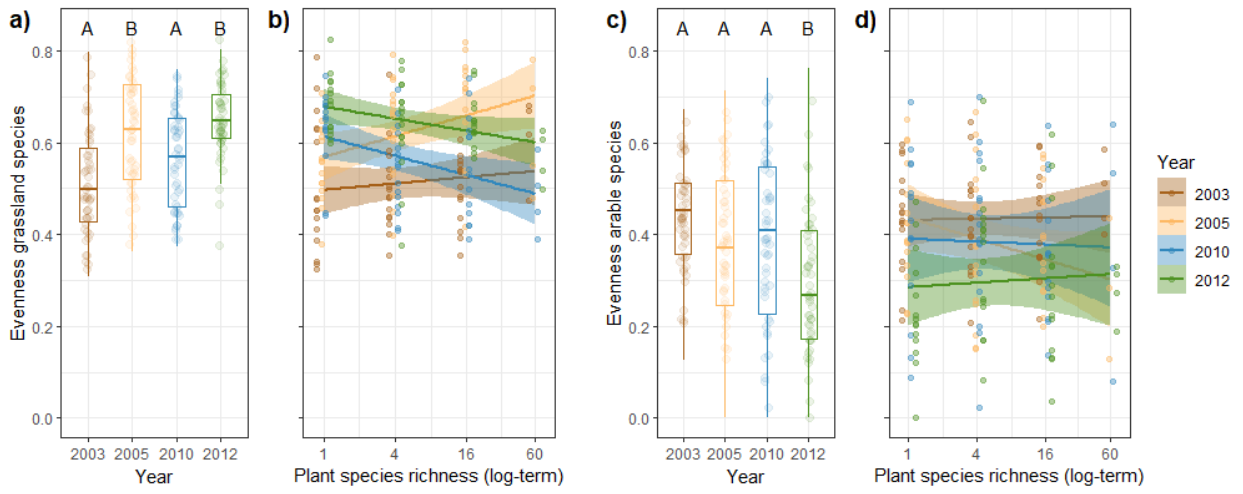

Figure S1: Evenness of a), b) grassland carabid beetle and c), d) arable carabid beetle at different years and as affected by plant species richness. Lines in boxes represent median, top and bottom of boxes represent first and third quartiles, and whiskers represent 1.5 interquartile range. Differences among the years are assessed based on estimated marginal means. Boxes with the same letters are not statistically different. Bands in regression plots represent the level of confidence interval (0.95). Dots represent partial residuals for "Year" and "Plant species richness (log-term).

Table S1: List of plant species of the pool of 60 Arrhenatherion grassland plant species, grouped according to their functional group[1].

Grasses	Legumes	Samll Herbs	Tall Herbs
Bromus erectus	Trifolium pratense	Taraxacum officinale agg.	Cardamine pratensis
Avenula pubescens	Trifolium repens	Veronica chamaedris	Galium mollugo agg.
Arrhenatherum elatius	Vicia cracca	Ranunculus repens	Daucus carota
Anthoxanthum odoratum	Lathyrus pratensis	Prunella vulgaris	Crepis biennis
Alopecurus pratensis	Trifolium fragiferum	Primula veris	Cirsium oleraceum
Festuca rubra	Lotus corniculatus	Plantago media	Centaurea jacea ssp. Jacea
Holcus lanatus	Medicago varia	Plantago lanceolata	Achillea millefolium
Festuca pratensis	Medicago lupulina	Leontodon hispidus	Carum carvi
Luzulus campestre	Trifolium hybridum	Leontodon autumnalis	Campanula patula
Dactylis glomerata	Trifolium campestre	Glechoma hederacea	Anthriscus sylvestris
Phleum pratense	Trifolium dubium	Ajuga reptans	Heracleum sphondylium
Cynosurus cristatus	Onobrychis viciifolia	Bellis perennis	Knautia arvensis
Poa pratense			Leucanthemum vulgare agg.
Poa trivialis			Pastinaca sativa
Trisetum flavescens			Pimpinella major
Bromus hordeaceus			Ranunculus acris
			Rumex acetosa
			Tragopogon pratensis
			Sanguisorba officinalis
			Geranium pratense

[1] Roscher, C. et al. The role of biodiversity for element cycling and trophic interactions: an experimental approach in a grassland community. Basic and Applied Ecology 5, 107-121 (2004).

Table S2: Carabid species, their annual abundance (N), characterized by their habitat preferences (g = grassland, a = Arable land, o = other habitat preferences) and feeding guilds (C = carbivor, P = phytophag, O = omnivor, NA = no information available).

Species	Habitat preferences	Feeding guilds	N in 2003	N in 2005	N in 2010	N in 2012	Total N
Acupalpus meridianus	g	C	1	0	1	2	4
Agonum muelleri	g	C	6	0	0	0	6
Amara aenea	g	P	161	361	479	1834	2835
Amara apricaria	g	P	1	0	0	0	1
Amara aulica	g	P	372	240	108	93	813
Amara bifrons	g	P	53	38	138	37	266
Amara communis	g	P	12	16	0	4	32
Amara consularis	g	P	7	0	0	0	7
Amara convexior	g	P	85	49	40	51	225
Amara equestris	g	P	5	174	46	70	295
Amara eurynota	o	P	2	0	0	0	2
Amara familiaris	g	P	8	4	16	25	53
Amara lunicollis	g	P	30	16	7	1	54
Amara nitida	g	P	58	726	114	162	1060
Amara ovata	a	P	18	7	1	0	26
Amara plebeja	g	P	69	1	3	1	74
Amara similata	g	P	303	8	0	3	314
Anchomenus dorsalis	g	C	135	74	293	321	823
Anisodactylus binotatus	a	C	247	9	6	1	263
Asaphidion flavipes	a	C	0	2	3	1	6
Asaphidion pallipes	o	C	0	0	5	4	9
Badister bullatus	g	C	6	8	35	38	87
Badister sodalis	o	C	1	0	0	2	3
Bembidion guttula	g	NA	0	0	0	1	1
Bembidion lampros	a	C	6	10	48	44	108
Bembidion lunulatum	o	C	0	1	1	0	2
Bembidion obtusum	g	NA	0	0	0	4	4
Bembidion properans	g	C	28	7	299	1086	1420
Bembidion quadrimaculatum	g	C	20	7	21	26	74
Brachinus crepitans	o	C	0	1	0	0	1
Brachinus explodens	o	C	14	158	100	59	331
Bradycellus csikii	o	C	2	0	0	0	2
Calathus cinctus	g	C	1	0	0	0	1
Calathus erratus	g	C	29	26	1	0	56
Calathus fuscipes	g	C	116	355	256	360	1087
Calathus melanocephalus	a	O	22	5	9	4	40

Carabus auratus	g	C	48	41	412	98	599
Carabus coriaceus	g	C	0	0	0	2	2
Carabus granulatus	a	C	15	4	0	0	19
Carabus nemoralis	o	C	39	195	30	8	272
Chlaenius nigricornis	o	C	1	0	0	0	1
Cicindela campestris	g	C	0	2	0	1	3
Clivina fossor	g	C	3	2	1	3	9
Diachromus germanus	o	O	14	0	1	0	15
Dolichus halensis	o	NA	0	0	0	1	1
Harpalus affinis	g	P	302	329	520	993	2144
Harpalus atratus	o	O	2	0	0	0	2
Harpalus dimidiatus	a	C	0	0	1	0	1
Harpalus distinguendus	a	O	960	88	22	11	1081
Harpalus latus	g	O	2	8	19	14	43
Harpalus luteicornis	g	NA	0	0	0	1	1
Harpalus rubripes	g	O	29	34	44	74	181
Harpalus rufipes	a	O	8792	1496	1066	1714	13068
Harpalus signaticornis	g	NA	0	0	0	2	2
Harpalus subcylindricus	o	C	0	0	1	0	1
Harpalus tardus	g	O	2	0	0	0	2
Leistus ferrugineus	g	C	27	42	8	1	78
Loricera pilicornis	g	C	172	11	2	3	188
Microlestes maurus	o	C	0	30	1022	756	1808
Microlestes minutulus	a	C	3	69	10	13	95
Nebria brevicollis	g	C	892	775	199	258	2124
Notiophilus aestuans	g	C	4	21	12	7	44
Notiophilus aquaticus	o	C	0	1	5	2	8
Notiophilus biguttatus	o	C	1	3	0	0	4
Notiophilus palustris	g	C	0	10	1	0	11
Oodes helopioides	g	NA	0	0	0	1	1
Ophonus ardosiacus	o	C	0	0	3	2	5
Ophonus azureus	o	O	3	18	73	108	202
Ophonus diffinis	o	C	0	0	1	0	1
Ophonus laticollis	g	NA	3	3	1	2	9
Ophonus melletii	o	C	0	0	2	0	2
Ophonus puncticeps	o	O	55	33	17	9	114
Ophonus rufibarbis	g	O	3	0	0	0	3
Ophonus schaubergerianus	g	C	0	0	32	110	142
Ophonus stictus	g	C	0	0	1	16	17
Platynus assimilis	g	NA	0	0	2	0	2

Poecilus cupreus	a	C	2964	202	126	200	3492
Poecilus versicolor	g	C	75	149	79	184	487
Pterostichus melanarius	g	O	5390	2946	4765	3714	16815
Pterostichus minor	o	C	0	1	0	0	1
Pterostichus niger	o	C	0	0	0	1	1
Pterostichus strenuus	o	C	0	0	1	2	3
Pterostichus vernalis	g	C	6	2	0	2	10
Stomis pumicatus	g	C	15	5	1	0	21
Syntomus truncatellus	g	C	0	0	2	18	20
Synuchus vivalis	g	C	4	0	6	10	20
Trechoblemus micros	o	NA	0	0	0	3	3
Trechus quadristriatus	g	C	24	31	6	10	71
**Sum arable**	a		13027	1892	1292	1988	18199
**Sum grass**	g		8507	6521	7970	9643	32641
**Sum others**	o		134	441	1262	957	2794
**Sum total**			21668	8854	10524	12588	53634

Table S3: Weather data for all sampling years, summarized for the winter period before sampling (Dec - Mar) and the vegetation period of sampling (Apr - Oct). Weather data were recorded at the weather station on the field site of the Jena Experiment (https://www.bgc-jena.mpg.de/wetter/).

Year	Season	Mean temp. (°C)	Precipitation (L m^{-2})	No. summer days (T_{max} > 25 °C)	No. hot days (T_{max} > 30 °C)	No. ice days (T_{max} < 0 °C)
2003	Winter	1.8	120.2	0	0	22
	Vegetation period	14.8	320.1	79	32	0
2005	Winter	1.8	117	0	0	18
	Vegetation period	14	274.1	45	11	0
2010	Winter	0.4	143.7	0	0	36
	Vegetation period	13.6	490	42	13	0
2012	Winter	2.8	136.5	0	0	16
	Vegetation period	13.9	352.3	45	12	0
