# Supplementary material for: Restoration of insect communities after land use change is shaped by plant diversity: a case study on carabid beetles (Carabidae)
Source: Sci Rep. 2023 Feb 7;13:2140. doi: 10.1038/s41598-023-28628-7 (PMC9905558; doi:10.1038/s41598-023-28628-7)
Supplement: Supplementary file 2 — Supplementary Information 2. [file 41598_2023_28628_MOESM2_ESM.pdf]

R Markdown: Restoration of insect communities after land use change is shaped by plant diversity – a case study on carabid beetles (*Carabidae*)

Markus Lange

2023-01-10

Effects of Time and Plant Species Richness on Total Carabid Abundance and Richness

Using Linear Mixed Models (LMM) and Estimated Marginal Means (EMM)

LMM: Carabid Abundance (N)

```
library(lme4)
```

```
## Loading required package: Matrix
```

```
library(emmeans)

#Abundance
m0_N<-lmer(N~1+
             (1|plot),REML=F,data=div_tab)
m01_N<-lmer(N~block+
              (1|plot),REML=F,data=div_tab)
m1_N<-lmer(N~block+factor(year)+
             (1|plot),REML=F,data=div_tab)
m2_N<-lmer(N~block+factor(year)+lg_psr+
             (1|plot),REML=F,data=div_tab)
m7_N<-lmer(N~block+factor(year)+lg_psr+
             factor(year)*lg_psr+
             (1|plot),REML=F,data=div_tab)
anova(m0_N,m01_N,m1_N,m2_N,m7_N)
```

```
## Data: div_tab
## Models:
## m0_N: N ~ 1 + (1 | plot)
## m01_N: N ~ block + (1 | plot)
## m1_N: N ~ block + factor(year) + (1 | plot)
## m2_N: N ~ block + factor(year) + lg_psr + (1 | plot)
## m7_N: N ~ block + factor(year) + lg_psr + factor(year) * lg_psr + (1 | plot)
##       npar    AIC    BIC  logLik deviance    Chisq Df Pr(>Chisq)
```

```
## m0_N     3 2529.1 2538.9 -1261.5   2523.1
## m01_N    6 2532.5 2552.2 -1260.3   2520.5   2.5459  3     0.4671
## m1_N     9 2402.1 2431.6 -1192.0   2384.1 136.4246  3     <2e-16 ***
## m2_N    10 2404.0 2436.8 -1192.0   2384.0   0.0977  1     0.7546
## m7_N    13 2407.6 2450.2 -1190.8   2381.6   2.4029  3     0.4931
## ---
## Signif. codes:  0 '***' 0.001 '**' 0.01 '*' 0.05 '.' 0.1 ' ' 1
```

```
if(requireNamespace("multcomp")) {
  m1_N_ph<-lmer(N~block+factor(year)+
                (1|plot),REML=T,data=caras_N)

  m1_N_ph.emm <- emmeans(m1_N_ph,  ~ factor(year))
  multcomp::cld(m1_N_ph.emm, alpha = 0.05, Letters = LETTERS)
}
```

EMM: Differences of N Over Time

```
##  year emmean   SE  df lower.CL upper.CL .group
##  2005    177 16.6 127      144      210  A
##  2010    220 16.9 131      187      253  AB
##  2012    263 16.9 131      230      296   B
##  2003    433 16.6 127      400      466    C
##
## Results are averaged over the levels of: block
## Degrees-of-freedom method: kenward-roger
## Confidence level used: 0.95
## P value adjustment: tukey method for comparing a family of 4 estimates
## significance level used: alpha = 0.05
## NOTE: If two or more means share the same grouping letter,
##       then we cannot show them to be different.
##       But we also did not show them to be the same.
```

LMM: Carabid Richness (S)

```
#Species Richness

m0_S<-lmer(S~1+
             (1|plot),REML=F,data=div_tab)
m01_S<-lmer(S~block+
              (1|plot),REML=F,data=div_tab)
m1_S<-lmer(S~block+factor(year)+
             (1|plot),REML=F,data=div_tab)
m2_S<-lmer(S~block+factor(year)+lg_psr+
             (1|plot),REML=F,data=div_tab)
m7_S<-lmer(S~block+factor(year)+lg_psr+
             factor(year)*lg_psr+
             (1|plot),REML=F,data=div_tab)
anova(m0_S,m01_S,m1_S,m2_S,m7_S)
```

```
## Data: div_tab
## Models:
## m0_S: S ~ 1 + (1 | plot)
## m01_S: S ~ block + (1 | plot)
## m1_S: S ~ block + factor(year) + (1 | plot)
## m2_S: S ~ block + factor(year) + lg_psr + (1 | plot)
## m7_S: S ~ block + factor(year) + lg_psr + factor(year) * lg_psr + (1 | plot)
##       npar    AIC    BIC  logLik deviance   Chisq Df Pr(>Chisq)
## m0_S     3 1028.0 1037.8 -511.00  1021.99
## m01_S    6 1026.6 1046.3 -507.32  1014.64  7.3550  3   0.061403 .
## m1_S     9 1030.7 1060.2 -506.37  1012.74  1.8969  3   0.594084
## m2_S    10 1030.7 1063.5 -505.35  1010.70  2.0472  1   0.152488
## m7_S    13 1024.5 1067.1 -499.25   998.49 12.2025  3   0.006721 **
## ---
## Signif. codes:  0 '***' 0.001 '**' 0.01 '*' 0.05 '.' 0.1 ' ' 1
```

```
if(requireNamespace("multcomp")) {
  m1_S_ph<-lmer(S~block+factor(year)+
              (1|plot),REML=T,data=div_tab)
  m1_S_ph.emm <- emmeans(m1_S_ph,  ~ factor(year))
  multcomp::cld(m1_S_ph.emm, alpha = 0.05, Letters = LETTERS)
}
```

EMM: Differences of S Over Time

```
##  year emmean    SE  df lower.CL upper.CL .group
##  2005   18.5 0.464 183     17.6     19.4  A
##  2012   18.6 0.474 184     17.7     19.6  A
##  2010   18.7 0.474 184     17.8     19.6  A
##  2003   19.3 0.464 183     18.4     20.2  A
##
## Results are averaged over the levels of: block
## Degrees-of-freedom method: kenward-roger
## Confidence level used: 0.95
## P value adjustment: tukey method for comparing a family of 4 estimates
## significance level used: alpha = 0.05
## NOTE: If two or more means share the same grouping letter,
##       then we cannot show them to be different.
##       But we also did not show them to be the same.
```

```
m7_S_emm2<-lmer(S~block+factor(year)+lg_psr+
             factor(year)*lg_psr+
             (1|plot),REML=T,data=div_tab)
emtrends(m7_S_emm2, pairwise ~ factor(year), var = "lg_psr")
```

EMM: Differences of Plant Species Effect on S Over Time

```
## $emtrends
##  year lg_psr.trend    SE  df lower.CL upper.CL
##  2003        1.780 0.983 178   -0.159    3.719
##  2005       -1.268 0.983 178   -3.207    0.671
##  2010       -0.278 1.008 179   -2.267    1.712
##  2012        2.925 1.008 179    0.935    4.915
##
## Results are averaged over the levels of: block
## Degrees-of-freedom method: kenward-roger
## Confidence level used: 0.95
##
## $contrasts
##  contrast            estimate   SE  df t.ratio p.value
##  year2003 - year2005    3.048 1.32 140   2.309  0.1007
##  year2003 - year2010    2.058 1.34 142   1.537  0.4185
##  year2003 - year2012   -1.145 1.34 142  -0.855  0.8279
##  year2005 - year2010   -0.991 1.34 142  -0.740  0.8809
##  year2005 - year2012   -4.193 1.34 142  -3.131  0.0112
##  year2010 - year2012   -3.203 1.35 140  -2.365  0.0888
##
## Results are averaged over the levels of: block
## Degrees-of-freedom method: kenward-roger
## P value adjustment: tukey method for comparing a family of 4 estimates
```

Effects of Time and Plant Species Richness on Relative Abundances and Richnesses of Grassland and Arable Land Species

LMM: Abundances of Grassland Carabids (N_G)

```
library(lme4)
m0_Ngr_perc<-lmer(N_grass_perc~1+
                      (1|plot),REML=F,data=caras_habitat)
```

```
## boundary (singular) fit: see help('isSingular')
```

```
m01_Ngr_perc<-lmer(N_grass_perc~block+
                       (1|plot),REML=F,data=caras_habitat)
```

```
## boundary (singular) fit: see help('isSingular')
```

```
m1_Ngr_perc<-lmer(N_grass_perc~block+factor(year)+
                      (1|plot),REML=F,data=caras_habitat)
m2_Ngr_perc<-lmer(N_grass_perc~block+factor(year)+lg_psr+
                      (1|plot),REML=F,data=caras_habitat)
m7_Ngr_perc<-lmer(N_grass_perc~block+factor(year)+lg_psr+
                      factor(year)*lg_psr+
                      (1|plot),REML=F,data=caras_habitat)
anova(m0_Ngr_perc,m01_Ngr_perc,m1_Ngr_perc,m2_Ngr_perc,m7_Ngr_perc)
```

```
## Data: caras_habitat
```

```
## Models:
## m0_Ngr_perc: N_grass_perc ~ 1 + (1 | plot)
## m01_Ngr_perc: N_grass_perc ~ block + (1 | plot)
## m1_Ngr_perc: N_grass_perc ~ block + factor(year) + (1 | plot)
## m2_Ngr_perc: N_grass_perc ~ block + factor(year) + lg_psr + (1 | plot)
## m7_Ngr_perc: N_grass_perc ~ block + factor(year) + lg_psr + factor(year) * lg_psr + (1 | plot)
##              npar     AIC     BIC  logLik deviance    Chisq Df Pr(>Chisq)
## m0_Ngr_perc     3 -114.63 -104.80  60.316  -120.63
## m01_Ngr_perc    6 -112.79  -93.12  62.394  -124.79   4.1562  3    0.24509
## m1_Ngr_perc     9 -384.70 -355.20 201.350  -402.70 277.9109  3    < 2e-16 ***
## m2_Ngr_perc    10 -382.86 -350.08 201.429  -402.86   0.1585  1    0.69053
## m7_Ngr_perc    13 -384.34 -341.73 205.172  -410.34   7.4862  3    0.05791 .
## ---
## Signif. codes:  0 '***' 0.001 '**' 0.01 '*' 0.05 '.' 0.1 ' ' 1
```

```
if(requireNamespace("multcomp")) {
  m1_Ngr_perc_ph<-lmer(N_grass_perc~block+factor(year)+
                      (1|plot),REML=T,data=caras_habitat)
  m1_Ngr_perc_ph.emm <- emmeans(m1_Ngr_perc_ph,  ~ factor(year))
  multcomp::cld(m1_Ngr_perc_ph.emm, alpha = 0.05, Letters = LETTERS)
}
```

EMM: Differences of N_G Over Time

```
##  year emmean     SE  df lower.CL upper.CL .group
##  2003  0.394 0.0126 180    0.369    0.418  A
##  2005  0.726 0.0126 180    0.701    0.751   B
##  2010  0.750 0.0128 181    0.724    0.775   B
##  2012  0.755 0.0128 181    0.730    0.781   B
##
## Results are averaged over the levels of: block
## Degrees-of-freedom method: kenward-roger
## Confidence level used: 0.95
## P value adjustment: tukey method for comparing a family of 4 estimates
## significance level used: alpha = 0.05
## NOTE: If two or more means share the same grouping letter,
##       then we cannot show them to be different.
##       But we also did not show them to be the same.
```

```
m7_N_emm2<-lmer(N~block+factor(year)+lg_psr+
             factor(year)*lg_psr+
             (1|plot),REML=T,data=div_tab)
emtrends(m7_N_emm2, pairwise ~ factor(year), var = "lg_psr")
```

EMM: Differences of Plant Species Effect on N_G Over Time

```
## $emtrends
##  year lg_psr.trend   SE  df lower.CL upper.CL
```

```
##  2003         6.39 36.3 124    -65.4     78.2
##  2005       -44.13 36.3 124   -115.9     27.7
##  2010        -3.49 37.0 128    -76.8     69.8
##  2012        10.95 37.0 128    -62.3     84.2
##
## Results are averaged over the levels of: block
## Degrees-of-freedom method: kenward-roger
## Confidence level used: 0.95
##
## $contrasts
##  contrast            estimate   SE  df t.ratio p.value
##  year2003 - year2005    50.52 40.1 140   1.259  0.5905
##  year2003 - year2010     9.88 40.8 141   0.242  0.9950
##  year2003 - year2012    -4.56 40.8 141  -0.112  0.9995
##  year2005 - year2010   -40.64 40.8 141  -0.996  0.7521
##  year2005 - year2012   -55.08 40.8 141  -1.350  0.5331
##  year2010 - year2012   -14.45 41.2 140  -0.351  0.9851
##
## Results are averaged over the levels of: block
## Degrees-of-freedom method: kenward-roger
## P value adjustment: tukey method for comparing a family of 4 estimates
```

LMM: Grassland Carabid Richness (S_G)

```
m0_grs_perc<-lmer(grassS_perc~1+
                    (1|plot),REML=F,data=caras_habitat_s)
m01_grs_perc<-lmer(grassS_perc~block+
                     (1|plot),REML=F,data=caras_habitat_s)
m1_grs_perc<-lmer(grassS_perc~block+factor(year)+
                    (1|plot),REML=F,data=caras_habitat_s)
m2_grs_perc<-lmer(grassS_perc~block+factor(year)+lg_psr+
                    (1|plot),REML=F,data=caras_habitat_s)
m7_grs_perc<-lmer(grassS_perc~block+factor(year)+lg_psr+
                    factor(year)*lg_psr+
                    (1|plot),REML=F,data=caras_habitat_s)
anova(m0_grs_perc,m01_grs_perc,m1_grs_perc,m2_grs_perc,m7_grs_perc)
```

```
## Data: caras_habitat_s
## Models:
## m0_grs_perc: grassS_perc ~ 1 + (1 | plot)
## m01_grs_perc: grassS_perc ~ block + (1 | plot)
## m1_grs_perc: grassS_perc ~ block + factor(year) + (1 | plot)
## m2_grs_perc: grassS_perc ~ block + factor(year) + lg_psr + (1 | plot)
## m7_grs_perc: grassS_perc ~ block + factor(year) + lg_psr + factor(year) * lg_psr + (1 | plot)
##              npar     AIC     BIC logLik deviance  Chisq Df Pr(>Chisq)
## m0_grs_perc     3 -546.86 -537.03 276.43  -552.86
## m01_grs_perc    6 -541.32 -521.65 276.66  -553.32 0.4571  3     0.9282
## m1_grs_perc     9 -539.05 -509.55 278.52  -557.05 3.7301  3     0.2921
## m2_grs_perc    10 -539.72 -506.94 279.86  -559.72 2.6695  1     0.1023
## m7_grs_perc    13 -543.66 -501.04 284.83  -569.66 9.9381  3     0.0191 *
## ---
## Signif. codes:  0 '***' 0.001 '**' 0.01 '*' 0.05 '.' 0.1 ' ' 1
```

```
if(requireNamespace("multcomp")) {
  m1_grs_perc_ph<-lmer(grassS_perc~block+factor(year)+
                    (1|plot),REML=T,data=caras_habitat_s)
  m1_grs_perc_ph.emm <- emmeans(m1_grs_perc_ph,  ~ factor(year))
  multcomp::cld(m1_grs_perc_ph.emm, alpha = 0.05, Letters = LETTERS)
}
```

EMM: differences over time of S_G

```
##  year emmean      SE  df lower.CL upper.CL .group
##  2005  0.669 0.00852 176    0.653    0.686  A
##  2010  0.671 0.00870 177    0.654    0.688  A
##  2003  0.683 0.00852 176    0.666    0.699  A
##  2012  0.688 0.00870 177    0.670    0.705  A
##
## Results are averaged over the levels of: block
## Degrees-of-freedom method: kenward-roger
## Confidence level used: 0.95
## P value adjustment: tukey method for comparing a family of 4 estimates
## significance level used: alpha = 0.05
## NOTE: If two or more means share the same grouping letter,
##       then we cannot show them to be different.
##       But we also did not show them to be the same.
```

```
m7_S_emm2<-lmer(S~block+factor(year)+lg_psr+
             factor(year)*lg_psr+
             (1|plot),REML=T,data=div_tab)
emtrends(m7_S_emm2, pairwise ~ factor(year), var = "lg_psr")
```

EMM: Differences of Plant Species Effect on S_G over time

```
## $emtrends
##  year lg_psr.trend    SE  df lower.CL upper.CL
##  2003        1.780 0.983 178   -0.159    3.719
##  2005       -1.268 0.983 178   -3.207    0.671
##  2010       -0.278 1.008 179   -2.267    1.712
##  2012        2.925 1.008 179    0.935    4.915
##
## Results are averaged over the levels of: block
## Degrees-of-freedom method: kenward-roger
## Confidence level used: 0.95
##
## $contrasts
##  contrast              estimate   SE  df t.ratio p.value
##  year2003 - year2005    3.048 1.32 140   2.309  0.1007
##  year2003 - year2010    2.058 1.34 142   1.537  0.4185
##  year2003 - year2012   -1.145 1.34 142  -0.855  0.8279
##  year2005 - year2010   -0.991 1.34 142  -0.740  0.8809
```

```
##  year2005 - year2012   -4.193 1.34 142  -3.131  0.0112
##  year2010 - year2012   -3.203 1.35 140  -2.365  0.0888
##
## Results are averaged over the levels of: block
## Degrees-of-freedom method: kenward-roger
## P value adjustment: tukey method for comparing a family of 4 estimates
```

LMM: Arable land carabid abundance (N_A)

```
m0_Narbl_perc<-lmer(N_arable_perc~1+
                 (1|plot),REML=F,data=caras_habitat)
```

```
## boundary (singular) fit: see help('isSingular')
```

```
m01_Narbl_perc<-lmer(N_arable_perc~block+
                  (1|plot),REML=F,data=caras_habitat)
```

```
## boundary (singular) fit: see help('isSingular')
```

```
m1_Narbl_perc<-lmer(N_arable_perc~block+factor(year)+
                 (1|plot),REML=F,data=caras_habitat)
m2_Narbl_perc<-lmer(N_arable_perc~block+factor(year)+lg_psr+
                 (1|plot),REML=F,data=caras_habitat)
m7_Narbl_perc<-lmer(N_arable_perc~block+factor(year)+lg_psr+
                 factor(year)*lg_psr+
                 (1|plot),REML=F,data=caras_habitat)
anova(m0_Narbl_perc,m01_Narbl_perc,m1_Narbl_perc,m2_Narbl_perc,m7_Narbl_perc)
```

```
## Data: caras_habitat
## Models:
## m0_Narbl_perc: N_arable_perc ~ 1 + (1 | plot)
## m01_Narbl_perc: N_arable_perc ~ block + (1 | plot)
## m1_Narbl_perc: N_arable_perc ~ block + factor(year) + (1 | plot)
## m2_Narbl_perc: N_arable_perc ~ block + factor(year) + lg_psr + (1 | plot)
## m7_Narbl_perc: N_arable_perc ~ block + factor(year) + lg_psr + factor(year) * lg_psr + (1 | plot)
##                npar     AIC     BIC  logLik deviance    Chisq Df Pr(>Chisq)
## m0_Narbl_perc     3  -55.62  -45.79  30.810   -61.62
## m01_Narbl_perc    6  -51.91  -32.24  31.957   -63.91   2.2930  3     0.5139
## m1_Narbl_perc     9 -442.57 -413.07 230.286  -460.57 396.6582  3     <2e-16 ***
## m2_Narbl_perc    10 -440.58 -407.80 230.289  -460.58   0.0060  1     0.9381
## m7_Narbl_perc    13 -439.42 -396.81 232.710  -465.42   4.8428  3     0.1837
## ---
## Signif. codes:  0 '***' 0.001 '**' 0.01 '*' 0.05 '.' 0.1 ' ' 1
```

```
if(requireNamespace("multcomp")) {
  m1_Narbl_perc_ph<-lmer(N_arable_perc~block+factor(year)+
                        (1|plot),REML=T,data=caras_habitat)
```

```
  m1_Narbl_perc_ph.emm <- emmeans(m1_Narbl_perc_ph,  ~ factor(year))
  multcomp::cld(m1_Narbl_perc_ph.emm, alpha = 0.05, Letters = LETTERS)
}
```

EMM: Differences of N_A Over Time

```
##  year emmean     SE  df lower.CL upper.CL .group
##  2010  0.121 0.0113 167   0.0986    0.143  A
##  2012  0.164 0.0113 167   0.1413    0.186   B
##  2005  0.225 0.0110 165   0.2030    0.247    C
##  2003  0.600 0.0110 165   0.5777    0.621     D
##
## Results are averaged over the levels of: block
## Degrees-of-freedom method: kenward-roger
## Confidence level used: 0.95
## P value adjustment: tukey method for comparing a family of 4 estimates
## significance level used: alpha = 0.05
## NOTE: If two or more means share the same grouping letter,
##       then we cannot show them to be different.
##       But we also did not show them to be the same.
```

LMM: Arable Land carabid Richness (S_A)

```
m0_arab_perc<-lmer(arableS_perc~1+
                     (1|plot),REML=F,data=caras_habitat_s)
```

```
## boundary (singular) fit: see help('isSingular')
```

```
m01_arab_perc<-lmer(arableS_perc~block+
                      (1|plot),REML=F,data=caras_habitat_s)
```

```
## boundary (singular) fit: see help('isSingular')
```

```
m1_arab_perc<-lmer(arableS_perc~block+factor(year)+
                     (1|plot),REML=F,data=caras_habitat_s)
m2_arab_perc<-lmer(arableS_perc~block+factor(year)+lg_psr+
                     (1|plot),REML=F,data=caras_habitat_s)
m7_arab_perc<-lmer(arableS_perc~block+factor(year)+lg_psr+
                     factor(year)*lg_psr+
                     (1|plot),REML=F,data=caras_habitat_s)
anova(m0_arab_perc,m01_arab_perc,m1_arab_perc,m2_arab_perc,m7_arab_perc)
```

```
## Data: caras_habitat_s
## Models:
## m0_arab_perc: arableS_perc ~ 1 + (1 | plot)
## m01_arab_perc: arableS_perc ~ block + (1 | plot)
## m1_arab_perc: arableS_perc ~ block + factor(year) + (1 | plot)
## m2_arab_perc: arableS_perc ~ block + factor(year) + lg_psr + (1 | plot)
## m7_arab_perc: arableS_perc ~ block + factor(year) + lg_psr + factor(year) * lg_psr + (1 | plot)
```

```
##                npar     AIC     BIC logLik deviance   Chisq Df Pr(>Chisq)
## m0_arab_perc     3 -592.06 -582.22 299.03  -598.06
## m01_arab_perc    6 -587.88 -568.21 299.94  -599.88  1.8195  3   0.610703
## m1_arab_perc     9 -679.41 -649.90 348.70  -697.41 97.5290  3  < 2.2e-16 ***
## m2_arab_perc    10 -685.73 -652.95 352.87  -705.73  8.3281  1   0.003904 **
## m7_arab_perc    13 -687.99 -645.37 356.99  -713.99  8.2558  3   0.041011 *
## ---
## Signif. codes:  0 '***' 0.001 '**' 0.01 '*' 0.05 '.' 0.1 ' ' 1
```

```
if(requireNamespace("multcomp")) {
  m1_arbl_perc_ph<-lmer(arableS_perc~block+factor(year)+
                      (1|plot),REML=T,data=caras_habitat_s)
  m1_arbl_perc_ph.emm <- emmeans(m1_arbl_perc_ph,  ~ factor(year))
  multcomp::cld(m1_arbl_perc_ph.emm, alpha = 0.05, Letters = LETTERS)
}
```

EMM: Differences of S_A Over Time

```
##  year emmean      SE  df lower.CL upper.CL .group
##  2012  0.178 0.00607 179    0.166    0.190  A
##  2010  0.190 0.00607 179    0.178    0.202  A
##  2005  0.214 0.00594 178    0.202    0.226   B
##  2003  0.262 0.00594 178    0.250    0.274    C
##
## Results are averaged over the levels of: block
## Degrees-of-freedom method: kenward-roger
## Confidence level used: 0.95
## P value adjustment: tukey method for comparing a family of 4 estimates
## significance level used: alpha = 0.05
## NOTE: If two or more means share the same grouping letter,
##       then we cannot show them to be different.
##       But we also did not show them to be the same.
```

```
m7_arabS_emm2<-lmer(arableS_perc~block+factor(year)+lg_psr+
                     factor(year)*lg_psr+
                     (1|plot),REML=T,data=caras_habitat_s)
emtrends(m7_arabS_emm2, pairwise ~ factor(year), var = "lg_psr")
```

EMM: Differences of Plant Species Effect on S_A over time

```
## $emtrends
##  year lg_psr.trend     SE  df lower.CL  upper.CL
##  2003      0.00815 0.0124 178  -0.0164  0.032717
##  2005     -0.02486 0.0124 178  -0.0494 -0.000299
##  2010     -0.03124 0.0128 179  -0.0564 -0.006025
##  2012     -0.03494 0.0128 179  -0.0601 -0.009727
##
## Results are averaged over the levels of: block
```

```
## Degrees-of-freedom method: kenward-roger
## Confidence level used: 0.95
##
## $contrasts
##  contrast            estimate     SE  df t.ratio p.value
##  year2003 - year2005  0.03302 0.0167 140   1.974  0.2026
##  year2003 - year2010  0.03939 0.0170 142   2.321  0.0980
##  year2003 - year2012  0.04309 0.0170 142   2.540  0.0582
##  year2005 - year2010  0.00637 0.0170 142   0.375  0.9819
##  year2005 - year2012  0.01007 0.0170 142   0.594  0.9338
##  year2010 - year2012  0.00370 0.0172 140   0.216  0.9964
##
## Results are averaged over the levels of: block
## Degrees-of-freedom method: kenward-roger
## P value adjustment: tukey method for comparing a family of 4 estimates
```

LMM: Evenness of Grassland Carabids ($_{G}$J)

```
m0_grs_ev<-lmer(J_grass~1+
                    (1|plot),REML=F,data=caras_habitat_s)
m01_grs_ev<-lmer(J_grass~block+
                     (1|plot),REML=F,data=caras_habitat_s)
m1_grs_ev<-lmer(J_grass~block+factor(year)+
                    (1|plot),REML=F,data=caras_habitat_s)
m2_grs_ev<-lmer(J_grass~block+factor(year)+lg_psr+
                    (1|plot),REML=F,data=caras_habitat_s)
m7_grs_ev<-lmer(J_grass~block+factor(year)+lg_psr+
                    factor(year)*lg_psr+
                    (1|plot),REML=F,data=caras_habitat_s)
anova(m0_grs_ev,m01_grs_ev,m1_grs_ev,m2_grs_ev,m7_grs_ev)
```

```
## Data: caras_habitat_s
## Models:
## m0_grs_ev: J_grass ~ 1 + (1 | plot)
## m01_grs_ev: J_grass ~ block + (1 | plot)
## m1_grs_ev: J_grass ~ block + factor(year) + (1 | plot)
## m2_grs_ev: J_grass ~ block + factor(year) + lg_psr + (1 | plot)
## m7_grs_ev: J_grass ~ block + factor(year) + lg_psr + factor(year) * lg_psr + (1 | plot)
##            npar     AIC     BIC logLik deviance   Chisq Df Pr(>Chisq)
## m0_grs_ev     3 -264.84 -255.00 135.42  -270.84
## m01_grs_ev    6 -260.25 -240.58 136.13  -272.25  1.4154  3     0.7019
## m1_grs_ev     9 -299.16 -269.66 158.58  -317.16 44.9065  3  9.686e-10 ***
## m2_grs_ev    10 -297.19 -264.41 158.60  -317.19  0.0349  1     0.8518
## m7_grs_ev    13 -312.67 -270.05 169.33  -338.67 21.4720  3  8.400e-05 ***
## ---
## Signif. codes:  0 '***' 0.001 '**' 0.01 '*' 0.05 '.' 0.1 ' ' 1
```

```
if(requireNamespace("multcomp")) {
  m1_grs_ev_ph<-lmer(J_grass~block+factor(year)+
```

```
                          (1|plot),REML=T,data=caras_habitat_s)
  m1_grs_ev_ph.emm <- emmeans(m1_grs_ev_ph,  ~ factor(year))
  multcomp::cld(m1_grs_ev_ph.emm, alpha = 0.05, Letters = LETTERS)
}
```

EMM: Differences of $_G$J Over Time

```
##  year emmean     SE  df lower.CL upper.CL .group
##  2003  0.512 0.0158 172    0.481    0.543  A
##  2010  0.565 0.0161 173    0.533    0.597  A
##  2005  0.619 0.0158 172    0.588    0.650   B
##  2012  0.649 0.0161 173    0.617    0.680   B
##
## Results are averaged over the levels of: block
## Degrees-of-freedom method: kenward-roger
## Confidence level used: 0.95
## P value adjustment: tukey method for comparing a family of 4 estimates
## significance level used: alpha = 0.05
## NOTE: If two or more means share the same grouping letter,
##       then we cannot show them to be different.
##       But we also did not show them to be the same.
```

```
m7_grs_ev_ph<-lmer(J_grass~block+factor(year)+lg_psr+
                               factor(year)*lg_psr+
                               (1|plot),REML=T,data=caras_habitat_s)
emtrends(m7_grs_ev_ph, pairwise ~ factor(year), var = "lg_psr")
```

EMM: Differences of Plant Species Effect on $_G$J Over Time

```
## $emtrends
##  year lg_psr.trend     SE  df lower.CL upper.CL
##  2003       0.0320 0.0332 157  -0.0336  0.09765
##  2005       0.0886 0.0332 157   0.0230  0.15421
##  2010      -0.0880 0.0340 160  -0.1552 -0.02074
##  2012      -0.0589 0.0340 160  -0.1262  0.00828
##
## Results are averaged over the levels of: block
## Degrees-of-freedom method: kenward-roger
## Confidence level used: 0.95
##
## $contrasts
##  contrast            estimate     SE  df t.ratio p.value
##  year2003 - year2005  -0.0566 0.0414 140  -1.367  0.5223
##  year2003 - year2010   0.1200 0.0420 142   2.854  0.0253
##  year2003 - year2012   0.0910 0.0420 142   2.164  0.1385
##  year2005 - year2010   0.1765 0.0420 142   4.199  0.0003
##  year2005 - year2012   0.1475 0.0420 142   3.509  0.0033
##  year2010 - year2012  -0.0290 0.0425 140  -0.683  0.9033
##
## Results are averaged over the levels of: block
```

```
## Degrees-of-freedom method: kenward-roger
## P value adjustment: tukey method for comparing a family of 4 estimates
```

LMM: Evenness ofArable Land Carabids ($_A$J)

```
m0_arb_ev<-lmer(J_arable~1+
                  (1|plot),REML=F,data=caras_habitat_s)
m01_arb_ev<-lmer(J_arable~block+
                   (1|plot),REML=F,data=caras_habitat_s)
m1_arb_ev<-lmer(J_arable~block+factor(year)+
                  (1|plot),REML=F,data=caras_habitat_s)
m2_arb_ev<-lmer(J_arable~block+factor(year)+lg_psr+
                  (1|plot),REML=F,data=caras_habitat_s)
m7_arb_ev<-lmer(J_arable~block+factor(year)+lg_psr+
                  factor(year)*lg_psr+
                  (1|plot),REML=F,data=caras_habitat_s)
anova(m0_arb_ev,m01_arb_ev,m1_arb_ev,m2_arb_ev,m7_arb_ev)
```

```
## Data: caras_habitat_s
## Models:
## m0_arb_ev: J_arable ~ 1 + (1 | plot)
## m01_arb_ev: J_arable ~ block + (1 | plot)
## m1_arb_ev: J_arable ~ block + factor(year) + (1 | plot)
## m2_arb_ev: J_arable ~ block + factor(year) + lg_psr + (1 | plot)
## m7_arb_ev: J_arable ~ block + factor(year) + lg_psr + factor(year) * lg_psr + (1 | plot)
##            npar     AIC      BIC logLik deviance   Chisq Df Pr(>Chisq)
## m0_arb_ev     3 -125.02 -115.182 65.508  -131.02
## m01_arb_ev    6 -120.06 -100.396 66.032  -132.06  1.0479  3  0.7896616
## m1_arb_ev     9 -133.77 -104.269 75.886  -151.77 19.7082  3  0.0001951 ***
## m2_arb_ev    10 -132.25  -99.465 76.123  -152.25  0.4740  1  0.4911697
## m7_arb_ev    13 -129.84  -87.225 77.920  -155.84  3.5940  3  0.3087754
## ---
## Signif. codes:  0 '***' 0.001 '**' 0.01 '*' 0.05 '.' 0.1 ' ' 1
```

```
if(requireNamespace("multcomp")) {
  m1_arb_ev_ph<-lmer(J_arable~block+factor(year)+
                       (1|plot),REML=T,data=caras_habitat_s)
  m1_arb_ev_ph.emm <- emmeans(m1_arb_ev_ph,  ~ factor(year))
  multcomp::cld(m1_arb_ev_ph.emm, alpha = 0.05, Letters = LETTERS)
}
```

EMM: Differences of $_A$J Over Time

```
##  year emmean     SE  df lower.CL upper.CL .group
##  2012  0.295 0.0247 168    0.246    0.344  A
##  2010  0.382 0.0247 168    0.333    0.430   B
##  2005  0.386 0.0242 166    0.338    0.433   B
##  2003  0.434 0.0242 166    0.386    0.482   B
##
```

```
## Results are averaged over the levels of: block
## Degrees-of-freedom method: kenward-roger
## Confidence level used: 0.95
## P value adjustment: tukey method for comparing a family of 4 estimates
## significance level used: alpha = 0.05
## NOTE: If two or more means share the same grouping letter,
##       then we cannot show them to be different.
##       But we also did not show them to be the same.
```

Carabid Community Composition

PCoA

```r
#pre-transformation of the species data
caras.h <- decostand(caras, "hellinger")
#Distance matrices
caras.h.bray <- vegdist(caras.h,method="bray",na.rm = T)

cara.pcoa <- cmdscale(caras.h.bray, k = 8, eig = TRUE, add=TRUE)
(cara.pcoa.env <- envfit(cara.pcoa, vars2,na.rm = TRUE))
```

```
##
## ***VECTORS
##
##               Dim1     Dim2     r2 Pr(>r)
## block_num -0.60349 -0.79737 0.0036  0.717
## lg_psr    -0.00353  0.99999 0.1257  0.001 ***
## year       0.94024 -0.34050 0.8652  0.001 ***
## ---
## Signif. codes:  0 '***' 0.001 '**' 0.01 '*' 0.05 '.' 0.1 ' ' 1
## Permutation: free
## Number of permutations: 999
```

PERMANOVAs

```r
#####PERMANOVA all years
adonis2(caras.h  ~ block+factor(year)*lg_psr, data=vars, permutations=999)
```

```
## Permutation test for adonis under reduced model
## Terms added sequentially (first to last)
## Permutation: free
## Number of permutations: 999
##
## adonis2(formula = caras.h ~ block + factor(year) * lg_psr, data = vars, permutations = 999)
##                     Df SumOfSqs      R2       F Pr(>F)
## block                4   1.0760 0.04935  4.6714  0.001 ***
## factor(year)         3   8.5547 0.39237 49.5203  0.001 ***
## lg_psr               1   0.7885 0.03617 13.6939  0.001 ***
## factor(year):lg_psr  3   0.5001 0.02294  2.8950  0.001 ***
```

```
## Residual            189  10.8834 0.49917
## Total               200  21.8028 1.00000
## ---
## Signif. codes:  0 '***' 0.001 '**' 0.01 '*' 0.05 '.' 0.1 ' ' 1
```

```
#####PERMANOVA individual years
adonis2(rel_caras03.h  ~ block+lg_psr, data=vars03, permutations=999)
```

```
## Permutation test for adonis under reduced model
## Terms added sequentially (first to last)
## Permutation: free
## Number of permutations: 999
##
## adonis2(formula = rel_caras03.h ~ block + lg_psr, data = vars03, permutations = 999)
##          Df SumOfSqs      R2      F Pr(>F)
## block     3  0.29180 0.11709 2.1671  0.001 ***
## lg_psr    1  0.18059 0.07246 4.0236  0.001 ***
## Residual 45  2.01975 0.81045
## Total    49  2.49214 1.00000
## ---
## Signif. codes:  0 '***' 0.001 '**' 0.01 '*' 0.05 '.' 0.1 ' ' 1
```

```
adonis2(rel_caras05.h  ~ block+lg_psr, data=vars05, permutations=999)
```

```
## Permutation test for adonis under reduced model
## Terms added sequentially (first to last)
## Permutation: free
## Number of permutations: 999
##
## adonis2(formula = rel_caras05.h ~ block + lg_psr, data = vars05, permutations = 999)
##          Df SumOfSqs      R2      F Pr(>F)
## block     3   0.4540 0.11275 2.1105  0.001 ***
## lg_psr    1   0.3459 0.08590 4.8239  0.001 ***
## Residual 45   3.2268 0.80135
## Total    49   4.0267 1.00000
## ---
## Signif. codes:  0 '***' 0.001 '**' 0.01 '*' 0.05 '.' 0.1 ' ' 1
```

```
adonis2(rel_caras10.h  ~ block+lg_psr, data=vars10, permutations=999)
```

```
## Permutation test for adonis under reduced model
## Terms added sequentially (first to last)
## Permutation: free
## Number of permutations: 999
##
## adonis2(formula = rel_caras10.h ~ block + lg_psr, data = vars10, permutations = 999)
##          Df SumOfSqs      R2      F Pr(>F)
## block     3  0.34107 0.11440 2.1791  0.002 **
## lg_psr    1  0.39688 0.13312 7.6071  0.001 ***
## Residual 43  2.24340 0.75248
## Total    47  2.98134 1.00000
## ---
## Signif. codes:  0 '***' 0.001 '**' 0.01 '*' 0.05 '.' 0.1 ' ' 1
```

```
adonis2(rel_caras12.h  ~ block+lg_psr, data=vars12, permutations=999)
```

```
## Permutation test for adonis under reduced model
## Terms added sequentially (first to last)
## Permutation: free
## Number of permutations: 999
##
## adonis2(formula = rel_caras12.h ~ block + lg_psr, data = vars12, permutations = 999)
##          Df SumOfSqs      R2      F Pr(>F)
## block     3   0.3319 0.10224 1.9262  0.002 **
## lg_psr    1   0.4447 0.13696 7.7408  0.001 ***
## Residual 43   2.4701 0.76080
## Total    47   3.2467 1.00000
## ---
## Signif. codes:  0 '***' 0.001 '**' 0.01 '*' 0.05 '.' 0.1 ' ' 1
```

TURNOVER

LMM: Turnover of Community composition

```
library(lme4)
library(emmeans)
m0_bray<-lmer(bray~1+
             (1|plot),REML=F,data=beta_main)
```

```
## boundary (singular) fit: see help('isSingular')
```

```
m01_bray<-lmer(bray~block+
              (1|plot),REML=F,data=beta_main)
```

```
## boundary (singular) fit: see help('isSingular')
```

```
m1_bray<-lmer(bray~block+factor(period)+
             (1|plot),REML=F,data=beta_main)
```

```
## boundary (singular) fit: see help('isSingular')
```

```
m2_bray<-lmer(bray~block+factor(period)+lg_psr+
             (1|plot),REML=F,data=beta_main)
```

```
## boundary (singular) fit: see help('isSingular')
```

```
m7_bray<-lmer(bray~block+factor(period)+lg_psr+
             factor(period)*lg_psr+
             (1|plot),REML=F,data=beta_main)
```

```
## boundary (singular) fit: see help('isSingular')
```

```r
anova(m0_bray,m01_bray,m1_bray,m2_bray,m7_bray)
```

```
## Data: beta_main
## Models:
## m0_bray: bray ~ 1 + (1 | plot)
## m01_bray: bray ~ block + (1 | plot)
## m1_bray: bray ~ block + factor(period) + (1 | plot)
## m2_bray: bray ~ block + factor(period) + lg_psr + (1 | plot)
## m7_bray: bray ~ block + factor(period) + lg_psr + factor(period) * lg_psr + (1 | plot)
##          npar     AIC     BIC  logLik deviance   Chisq Df Pr(>Chisq)
## m0_bray     3 -170.24 -161.28  88.118  -176.24
## m01_bray    6 -168.36 -150.46  90.179  -180.36  4.1221  3   0.248577
## m1_bray     8 -239.68 -215.81 127.842  -255.68 75.3254  2  < 2.2e-16 ***
## m2_bray     9 -238.46 -211.61 128.229  -256.46  0.7742  1   0.378915
## m7_bray    11 -244.51 -211.69 133.254  -266.51 10.0507  2   0.006569 **
## ---
## Signif. codes:  0 '***' 0.001 '**' 0.01 '*' 0.05 '.' 0.1 ' ' 1
```

```r
if(requireNamespace("multcomp")) {
  m1_bray.emm<-lmer(bray~block+factor(period)+
             (1|plot),REML=T,data=beta_main)
  m1_bray.emm <- emmeans(m1_bray.emm,  ~ factor(period))
  multcomp::cld(m1_bray.emm, alpha = 0.05, Letters = LETTERS)
}
```

EMM: Community Turnover Over Time

```
## boundary (singular) fit: see help('isSingular')
```

```
##  period emmean     SE  df lower.CL upper.CL .group
##       3  0.422 0.0149 140    0.393    0.452  A
##       2  0.520 0.0149 140    0.491    0.550   B
##       1  0.625 0.0146 140    0.596    0.653    C
##
## Results are averaged over the levels of: block
## Degrees-of-freedom method: kenward-roger
## Confidence level used: 0.95
## P value adjustment: tukey method for comparing a family of 3 estimates
## significance level used: alpha = 0.05
## NOTE: If two or more means share the same grouping letter,
##       then we cannot show them to be different.
##       But we also did not show them to be the same.
```

```r
m7_bray.emm<-lmer(bray~block+factor(period)+lg_psr+
             factor(period)*lg_psr+
             (1|plot),REML=T,data=beta_main)
```

EMM: Differences of Plant Species Effect on Community Tur Over Time

```
## boundary (singular) fit: see help('isSingular')
```

```
emtrends(m7_bray.emm, pairwise ~ factor(period), var = "lg_psr")
```

```
## $emtrends
##  period lg_psr.trend     SE  df lower.CL upper.CL
##       1       0.0733 0.0308 137   0.0125  0.13419
##       2       0.0329 0.0316 137  -0.0296  0.09536
##       3      -0.0614 0.0316 137  -0.1239  0.00111
##
## Results are averaged over the levels of: block
## Degrees-of-freedom method: kenward-roger
## Confidence level used: 0.95
##
## $contrasts
##  contrast          estimate     SE   df t.ratio p.value
##  period1 - period2   0.0405 0.0441 94.5   0.918  0.6306
##  period1 - period3   0.1347 0.0441 94.5   3.055  0.0082
##  period2 - period3   0.0943 0.0446 92.8   2.111  0.0931
##
## Results are averaged over the levels of: block
## Degrees-of-freedom method: kenward-roger
## P value adjustment: tukey method for comparing a family of 3 estimates
```
